# MRI Correlates of Parkinson's Disease Progression: A Voxel Based Morphometry Study

**DOI:** 10.1155/2015/378032

**Published:** 2015-01-06

**Authors:** Valentina Fioravanti, Francesca Benuzzi, Luca Codeluppi, Sara Contardi, Francesco Cavallieri, Paolo Nichelli, Franco Valzania

**Affiliations:** Department of Neuroscience, Nuovo Ospedale Civile S. Agostino-Estense, University of Modena and Reggio Emilia, Viale Giardini 1355, 41126 Modena, Italy

## Abstract

We investigated structural brain differences between a group of early-mild PD patients at different phases of the disease and healthy subjects using voxel-based morphometry (VBM). 20 mild PD patients compared to 15 healthy at baseline and after 2 years of follow-up. VBM is a fully automated technique, which allows the identification of regional differences in the gray matter enabling an objective analysis of the whole brain between groups of subjects. With respect to controls, PD patients exhibited decreased GM volumes in right putamen and right parietal cortex. After 2 years of disease, the same patients confirmed GM loss in the putamen and parietal cortex; a significant difference was also observed in the area of pedunculopontine nucleus (PPN) and in the mesencephalic locomotor region (MLR). PD is associated with brain morphological changes in cortical and subcortical structures. The first regions to be affected in PD seem to be the parietal cortex and the putamen. A third structure that undergoes atrophy is the part of the inferior-posterior midbrain, attributable to the PPN and MLR. Our findings provide new insight into the brain involvement in PD and could contribute to a better understanding of the sequence of events occurring in these patients.

## 1. Introduction

Neuropathological studies in Parkinson's disease (PD) have proposed a six-phase progression system, indicating a growing and predictable sequence of brain pathology related to neurological deficits presented by patients at different stages of the disease [1]. However this classification often does not timely correlate with clinical severity or with onset of specific aspects of the symptomatology, such as postural or cognitive symptoms [2].

Only few studies investigated PD patients with short-disease duration. These studies reported no changes [3], lateral and medial frontal atrophy [4, 5], hippocampal/amygdala atrophy [4, 6, 7], and left cerebellum atrophy [8]. The purpose of this study was to identify cortical and subcortical structural changes at an early stage of PD and to evaluate their progression over 2 years.

The longitudinal evaluation of brain atrophy in patients with PD has been studied only in few studies with contradictory results. In fact, while Ramírez-Ruiz and collaborators showed the presence of a reduction of gray matter in several cortical areas [9], Brenneis and collaborators comparing a group of patients suffering from PD and a group of patients with multisystem atrophy (MSA-P) found a progression of the atrophy only in the group of patients with MSA-P, but not in patients with PD [10].

This lack of consistency is due to several factors in between-group design (e.g., number of samples, duration of the disease, variability in symptom characteristics, severity of illness, etc.). In light of this limitation, we opted for a within-group design to explore the effect of progression of the disease on brain atrophy in a group of PD patients.

## 2. Methods

### 2.1. Subjects

A group of 25 patients with PD were recruited at the Ambulatory of Movement Disorders at the Nuovo Ospedale Sant'Agostino-Estense (Modena). Fifteen healthy volunteers (HV), matched for age and gender, served as controls.

At baseline, all subjects underwent magnetic resonance imaging (MRI) scan, physical examination, and neuropsychological assessment. Those with a poor quality MRI scan or evidence of previous stroke were not included.

In summary, 20 PD patients (mean age = 63 yrs; 12 males and 8 females) and 15 age, gender, and education matched HV (mean age = 65 yrs; 8 males and 7 females) were included in the imaging analyses (Table 1).

Patients were diagnosed according to the UK Brain Bank criteria [11]. All patients used dopaminergic medication (levodopa or dopamine agonists). Disease severity was assessed using the Hoehn and Yahr stages and Unified Parkinson's Disease Rating Scale (UPDRS) in OFF-drug condition (at least 18 h following intake of the last dose of levodopa and 36 h after other antiparkinsonism medications). Patients were reassessed clinically and MRI after 2 years (mean = 25.5 ± 4.9 months).

The study was approved by the local ethics committee and written informed consent was obtained from participants prior to the experiment according to the Declaration of Helsinki.

### 2.2. MRI Acquisition

Scanning was performed at the Department of Neuroscience-Baggiovara, Hospital of Modena University. Three-dimensional (3D) T1-weighted MRI images were acquired using a 3 Tesla Philips Intera MRI scanner. A SPGR pulse sequence (echo time (TE) = 4.6, repetition time (TR) = 9.9 ms) was used. One hundred seventy contiguous sagittal slices were acquired (voxel size = 1 × 1 × 1 mm) and field of view was 240 mm with a matrix size of 256 × 256 × 170.

A T2-weighted axial scan and a coronal fluid attenuated inversion recovery (FLAIR) scan were also acquired to have a better definition of possible vascular damage.

### 2.3. Voxel-Based Morphometry (VBM) Analyses

The optimized VBM protocol was implemented within Matlab 7.1 (Mathworks, Natick, Mass.) through Statistical Parametric Mapping 8 (SPM8 Wellcome Trust Centre for Neuroimaging, London UK).

#### 2.3.1. Preprocessing

Preprocessing was performed using VBM8 (http://dbm.neuro.uni-jena.de/vbm/). This is an improved version of the standard SPM-VBM procedure described in detail elsewhere [12] and already used in previous studies [12, 13]. In brief, VBM8 comprised several processes: normalization, segmentation, modulation, and smoothing. The normalization step put the individual images in a common space: images were spatially normalized to a widely used T1-weighted MRI template in stereotaxic space, the Montreal Neurological Institute/International Consortium for Brain Mapping (MNI/ICBM) 152 standard. In this step, voxel size was resampled to 1.5 × 1.5 × 1.5 mm. Then images were segmented in GM, white matter (WM), and cerebrospinal fluid (CSF) volumes. Volumes were then modulated with Jacobian determinants. Modulation involves scaling by the amount of contraction, so that the total amount of GM in the modulated GM volumes remains the same, as it would be in the original images. Finally, segments were smoothed using a 12 × 12 × 12 kernel.

#### 2.3.2. Whole Brain Analysis

Total GM volumes of normalized-modulated images were compared between PD-baseline versus healthy subjects and PD-follow-up versus healthy subjects using a two-sample *t*-test. Since we had to compare the same group (patients at baseline and follow-up; dependent samples) with controls (independent samples), a single ANOVA could not be performed so we decided to evaluate the difference with separate *t*-test for independent samples. For the *t*-test the statistical threshold was set at *P* ≤ 0.001 uncorrected for multiple comparison. The extended threshold was differently set at baseline and follow-up analysis according to SPM approach. For the small volume correction (SVC) the threshold was set at *P* < 0.05 with familywise error (FWE) correction [13].

In the *t*-test we used age and TIV (total intracranial volume) as covariates of no interest. In addition, separate correlation analyses were run on the PD data using the following clinical scores: Hoehn and Yahr stages UPDRS scores and disease duration and therapy (levodopa equivalent dose).

The coordinates of significant voxels were converted from MNI space [14] to Talairach and Tournoux coordinates [15] using a Matlab function developed by M. Brett (mni2tal http://imaging.mrc-cbu.cam.ac.uk/imaging/MniTalairach). Subcortical anatomical regions were identified using the stereotactic atlas of Schaltenbrand-Wahren [16] second edition and the Nieuwenhuys-Voogd-Van Huijzen atlas [17].

## 3. Results

### 3.1. Baseline

**Figure 1:**
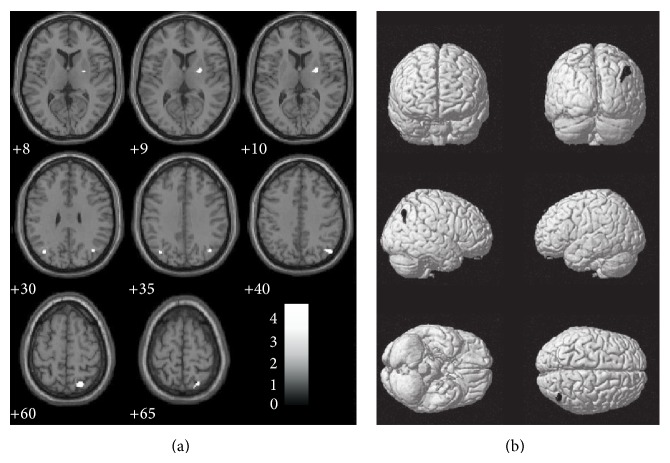
Surface rendering (a) and sagittal sections (b) showing the grey matter reduction in PD patients with respect to controls at baseline (test). Numbers below each sagittal slice represent the *x* coordinate in MNI space. Clusters are superimposed on the MNI template implemented in SPM8 (*P* = 0.001 uncorrected; *k* > 241 voxels).

We first tested the GM differences between PD patients and HV at baseline. The PD group showed decreased GM volume distributed in the right hemisphere, especially in the precuneus and in the parietal lobe (BA 39, BA 40, and BA 7; Table 2 and Figure 1). Additional GM loss was also identified in right putamen. At a less conservative threshold a symmetrical cluster has been observed also in the left parietal lobe.

No significant correlation with clinical parameters was found.

No significant increased GM volume in PD patients relative to controls was found.

### 3.2. Follow-Up Study

**Figure 2:**
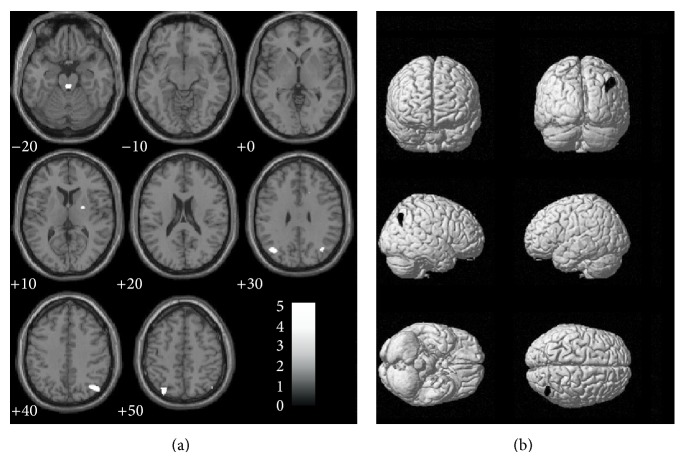
Surface rendering (a) and sagittal sections (b) showing the grey matter reduction in PD patients with respect to controls at retest. Numbers below each sagittal slice represent the *x* coordinate in MNI space. Clusters are superimposed on the MNI template implemented in SPM8 (*P* = 0.001 uncorrected; *k* > 223 voxels).

The comparison between PD patient after two years of follow-up and HV revealed that the PD group showed the same regions of GM loss found at baseline, but wider, involving the right parietal lobe (BA 39, BA 40, BA 7, and BA 31) and the right putamen (Table 3 and Figure 2). As observed before, a symmetrical not significant decrease of the gray matter was found in the left parietal lobe. Moreover, an additional GM loss cluster was found in the midbrain, in a region identified as the* mesencephalic locomotor region* (MLR, Figure 2). A similar cluster could be detected at baseline at a less conservative threshold. No significant correlation with UPDRS motor score (considered as a whole, and for specific postural control and path items) was found (UPDRS item 29 = 1.0 ± 0.8 at follow-up; UPDRS item 30 score of 0.9 ± 0.3 at baseline and 1.0 ± 0.4 at follow-up).

## 4. Discussion

We investigated structural brain differences between groups of healthy subjects and patients with PD using VBM. We evaluated patients at baseline and after two years of disease progression in OFF condition, after a pharmacological washout. The VBM analysis at baseline showed areas of GM loss in the right hemisphere, in particular in the putamen and parietal lobe. These regional atrophies were enlarged after two years of follow-up, where a new cluster in midbrain was also noted.

### 4.1. Putamen

Structural and functional alterations of the putamen have been widely documented in literature, using different methods. Péran et al. have shown the presence of alterations in the mean diffusivity in the striatum and in particular in the right putamen [18] in a group of thirty patients with PD. A dopamine binding transporter loss in this region has been found and neuropathological studies confirmed the presence of microscopic structural alterations such as deposition of Lewy bodies and accumulation of neurofibrillary in the same structures [19]. Molecular studies have recently confirmed the presence of a striatal progressive denervation and atrophy caused by the decrease in dopaminergic afferences due to neuronal loss in the substantia nigra [20].

The GM loss in the putamen in patients at mild to moderate stages of the disease is expected because a significant degeneration of dopaminergic nigrostriatal system precedes the onset of motor symptoms of PD. The right localization did not correlate with the onset side of symptoms nor with the handedness. Although apparently conflicting with data, PD symptoms seem to emerge more often on the dominant hand-side [21]. On the contrary, atrophy does not necessarily depend on nigrostriatal degeneration, which is usually more severe contralaterally to the more affected side. Moreover, it is known that the precentral motor cortex projects to the putamen and to other structures of the basal ganglia of both sides [22]. Therefore, this asymmetry may be related to the complex bilateral connections in the motor system that does not fulfill the side prevalence of clinical signs.

### 4.2. Parietal Cortex

The atrophy was found in the right parietal regions, in particular in the precuneus (PC) and in the angular gyrus (AG). This GM loss was wider in the follow-up analysis, in agreement with the disease progression. Several studies have shown the presence of atrophy in these parietal areas in PD patients [23, 24], without consistent differences between the two hemispheres. Functional and structural studies emphasized the central role of PC in a wide spectrum of abilities and it participates to the “default mode network” (DMN) a specific, anatomically defined brain system preferentially active when individuals are not focused on the external environment but participate in internal modes of cognition [25].

PC is selectively connected with other parietal structures (parietal caudal operculum and parietal lobules) for the processing of visuospatial information [26–28] and to the premotor cortex [29, 30], playing a central role in the visual coordination of hand and reaching movements [31].

Subcortical connections of PC are also relevant. They seem to come from the BA 31 region, which is a cortical transition zone from the medial parietal areas to the posterior cingulate and presents an apparent shift in cytoarchitecture from parietal isocortex to limbic cortex [32]. These projections reach several thalamic nuclei, the dorsolateral caudate nucleus, the putamen, the zona incerta, and, mainly, the brainstem structures such as the pretectal area, the superior colliculus, and the nucleus reticularis tegmenti pontis [33]. This last pathway, which brings together fibers coming from IPC, is functionally connected to the retinotectal visual system (RTVS), an ancient and subconscious visual network processing several visual parameters, as quality of oculomotor movements, contrast and colour discrimination, early impaired in PD and strongly involved in the motor behaviour [34].

The AG, particularly on the right side, is involved in higher-order aspects of motor control: the awareness that an intended action is consistent with movement consequences and the awareness of the authorship of the action (called “sense of agency”). Specifically, this region seems to process discrepancies between intended action and movement consequences in such a way that these will be consciously detected by the subject, ability that seems to be compromised in PD patients [35].

The structures of the parietal cortex play a central role in the networks which underlie both sustained attention on current task goals and various forms of response to salient stimuli in the environment to adapt to changing circumstances. In humans, the rostral part seems to be involved in motor planning and action-related functions and it is a part of the human mirror neuron system [36–39]. The right caudal parietal cortex is involved in spatial and nonspatial attention as well as motor preparation [40, 41], while left caudal parietal cortex is active during language-related tasks [42, 43]. This hemispheric specialization could explain prevalent atrophy observed in the right side because visuospatial functions, unlike linguistic ones, are often impaired in early PD [44].

Therefore, GM atrophy of the parietal structures observed in our early PD patients can be interpreted as the neuroanatomical correlate of visuospatial symptoms which we know to start early and often during the premotor phase.

### 4.3. Midbrain

At follow-up, an additional region of decreased grey volume was found in the midbrain. A similar cluster could be detected at baseline at a less conservative threshold. This cluster was located in the posterior region of the midbrain corresponding to the MLR of cat [16]; in humans it includes the pedunculopontine nucleus (PPN), the cuneiform nucleus, the periaqueductal gray, and the locus coeruleus [45, 46]. It receives inputs from basal ganglia, limbic system and supplementary and premotor cortex and it sends its main efferents to the basal ganglia, the brainstem, and the spinal cord [47].

With respect to the cellular density two main subdivisions of the mammals PPN have been recognized: a pars compacta of PPN (PPNc), constituted by cholinergic neurons; a pars dissipata (PPNd), containing a large proportion of glutaminergic neurons [48, 49].

Animal studies revealed a fundamental role of this structure in supraspinal locomotor circuit implicated in control of posture and gait, in particular for the rhythm and fluidity of step. In the decerebrate cat it has been shown [50] that 50 Hz stimulation of the MLR (corresponding to human PPNd) increased muscle tone and induced alternating hind limb stepping movements, enabling locomotor movements when the treadmill started to move. In the same experiment stimulation of PPN (corresponding to human PPNc) decreased muscle tone.

Anatomical and neurophysiological human studies confirmed such roles of MLR/PPN, in particular in the modulation of posture and gait initiation [47]. In a recent fMRI study on healthy subjects [51], mental imagery of stance and locomotion induced activation of cortical and subcortical areas including cerebellum, PPN, and cuneiform nucleus. This cluster resembled the MLR observed in the cat and demonstrated its similarity with the human locomotor network.

Hathout and Bhidayasiri [52] described three patients with ischemic lesions in the posterior midbrain tegmentum, corresponding to the cluster observed in our study. In addition to ataxia and vertical gaze palsy, probably related to the superior cerebellar pathways (CPS) and to third and fourth nerve impairment, all patients presented a start of hesitation and short and irregular steps without other motor parkinsonian signs.

We know that some axial symptoms in advanced PD, like a beginning hesitation, loss of steps rhythmicity, festination, and freezing, may be not dopa-responder and may depend on the involvement of cholinergic mesencephalic centers. In patients with progressive supranuclear palsy, PD, and Parkinson-Dementia, neuropathologic studies showed a strong loss of cholinergic neurons of PPN, correlating with the severity of disease [47, 53, 54]. Based on these observations some authors choose PPN as target of deep brain stimulation (DBS) in PD improving gait and other axial symptoms [55, 56]. Studying this model, Ballanger and collaborators showed that unilateral PPN-DBS increased rCBF in different subcortical area interconnected with PPN especially in cerebellum, thalamus, and a brainstem region corresponding to MLR [57].

In a recent VBM-fMRI study Snijders et al. [58] showed the presence, in PD patients with freezing of gait compared with nonfreezers, of an area of atrophy in a small posterior area of the midbrain corresponding to the MLR. Moreover, in the same patients, an fMRI study of motor imagery has shown an area of functional activation in the MLR, probably due to loss of balance between excitatory and inhibitory neurons in attempting to support gait planning and execution [58].

Our study confirmed the presence of GM atrophy in MLR. It is important to underline that, differently to Snijders and collaborators, we observed these data also in mild/moderate stage of PD, in which no patient had a clinically relevant gait or postural disorder. These data suggest an early impairment of MLR/PPN in PD and its specific role in the pathogenesis of postural and gait disorders.

## 5. Conclusions

The classical theory of the PD evolution developed by Braak did not receive convincing and univocal support by the neuroimaging techniques. A recent point of view [59] opposes the fixed temporal ordering of the caudal-cephalic propagation patterns of pathological lesions proposed by Braak et al. This new theory confers a role to the “archaic neural networks” consisting of parts of basal ganglia, midbrain, and parietal regions. An early frailty of this system could produce disrupted automatic gait control, olfactory and visual deficits, impaired emotional face recognition, and REM sleep behavior disorder that are the premotor core of PD. Our data, which show an early atrophy of putamen, parietal cortex and MLR, structures large enough to detect the atrophy, seem to support this hypothesis.

Although the small sample size prevents correcting the results for multiple comparison, our study shows that structural MRI measurement methods could be used to identify the presence of brain atrophy in specific regions known to be associated with PD pathogenesis, also before the occurrence of clinical symptoms. Furthermore this study has highlighted a progression of atrophy correlated with clinical worsening of patients. We hope that our results will shed light on the neuropathological progression of the PD pathology.

## Figures and Tables

**Table 1:** Sociodemographic and clinical characteristics of the two study groups. Values denote mean (± standard deviation) or numbers of subjects.

	PD patients *N* = 20	Controls *N* = 15
*Sociodemographics *		
Age (years)	60.5 (±7.7)	64.6 (±4.8)
Sex (male/female)	12/8	8/7
Education (years)	9.75 (±3.76)	11.3 (±3.19)
*Clinical characteristics *		
Duration of illness (years)	5.17 (±4.14)	
Age at diagnosis (years)	55.20 (±8.44)	
Baseline (test) OFF-drug		
UPDRS I	2.50 (±1.84)	
UPDRS II	8.55 (±3.88)	
UPDRS III	26.6 (±7.12)	
H&Y scale	2.42 (±0.25)	
Retest OFF-drug		
UPDRS I	2.61 (±1.50)	
UPDRS II	9.76 (±5.21)	
UPDRS III	22.46 (±6.02)	
H&Y scale	2.71 (±0.26)	
Equivalent dose of dopaminergic drug		
Test (mg)	473.5 (±238.0)	
Retest (mg)	650.3 (±272.9)	

**Table 2:** Grey matter reduction in PD patients with respect to controls at baseline (test).

	Cluster *k*	Voxel *Z*	Level *P*	MNI coord. *x*, *y*, *z* (mm)
R, parietal lobe (BA 40)	257	3.37	<0.001	48, −72, 45
R, angular gyrus (BA 39); R, precuneus (BA 31)				
R, inferior parietal lobule (BA 7)				39, −65, 32

R, putamen^*^	57	3.77	<0.05	26, −3, 9

*P* < 0.001 uncorrected for multiple comparisons (voxel level *k* > 241 voxels).

^*^Correction svc *P* = 0.05 (FWE).

L = left and R = right. BA = Brodmann area.

**Table 3:** Grey matter reduction in PD patients with respect to controls at retest.

	Cluster *k*	Voxel *Z*	Level *P*	MNI coord. *x*, *y*, *z* (mm)
R, parietal lobe (BA 40)	449	3.56	<0.001	48, −72, 46
R, angular gyrus (BA 39); R, precuneus (BA 31)				
R, inferior parietal lobule (BA 7)				
R, superior occipital gyrus (BA 19)		3.34	<0.001	39, −69, 31

R, putamen^*^	25	3.47	<0.05	24, −3, 10

Midbrain^*^	176	3.53	<0.05	2, −27, −17

*P* < 0.001 uncorrected for multiple comparisons (voxel level *k* > 241 voxels).

^*^Correction SVC *P* = 0.05 (FWE).

L = left and R = right. BA = Brodmann area.
